# Molecular Epidemiology of NDM-Producing *Escherichia coli* Isolates in Croatia from March 2023 to March 2024

**DOI:** 10.3390/microorganisms14040909

**Published:** 2026-04-17

**Authors:** Josip Ujević, Marko Jelić, Arjana Tambić Andrašević, Iva Butić

**Affiliations:** 1 Department of Clinical Microbiology, University Hospital for Infectious Diseases “Dr. Fran Mihaljević”, 10000 Zagreb, Croatia; jujevic@bfm.hr (J.U.); atambic@bfm.hr (A.T.A.); ibutic@bfm.hr (I.B.); 2 Dental School of Medicine, University of Zagreb, 10000 Zagreb, Croatia

**Keywords:** *E. coli*, NDM, Croatia, surveillance

## Abstract

In 2023, the European Centre for Disease Prevention and Control surveillance report highlighted an increasing number of carbapenem-resistant *Escherichia coli* isolates carrying the less common *bla*_NDM-5_ variant in Europe. The aim of this study was to investigate the molecular epidemiology of NDM-producing (New Delhi metallo-β-lactamase) *E. coli* isolates collected in Croatia over a one-year period. A total of 160 carbapenemase-producing *E. coli* isolates were reported through national surveillance in Croatia between March 2023 and March 2024. Whole-genome sequencing was performed on 22 NDM-producing *E. coli* isolates. Phylogenetic analysis identified 17 sequence types, indicating high diversity and polyclonal spread. High variability in resistome profiles and co-occurrence of resistance genes across multiple antimicrobial classes indicate multidrug resistance. The predominant *bla_NDM_* variant was *bla*_NDM-1_ (77.27%), followed by *bla*_NDM-5_ (22.73%). Co-occurrence of *bla_NDM_* with extended-spectrum β-lactamase (ESBL) encoding genes was detected in 12/22 isolates (54.55%). Plasmid analysis identified 22 different replicon types, with IncFII (54.54%) and IncA/C2 (45.45%) being the most frequent. Our findings provide insights into the molecular epidemiology of NDM-producing *E. coli* at the national level, highlighting the presence of the *bla*_NDM-5_ variant. These results emphasize the need for genomic surveillance and strengthened infection control strategies to better understand and limit its spread.

## 1. Introduction

Antimicrobial resistance (AMR) is a growing global threat, contributing to an estimated 1.27 million deaths in 2019 attributable to bacterial resistance [1,2]. The rise of multidrug-resistant organisms (MDRO), particularly Gram-negative bacteria, is complicating the treatment of common infections and placing a significant burden on healthcare systems [3,4]. Among the most concerning pathogens are *Escherichia coli* and other Enterobacterales, which are becoming more resistant to last-resort antibiotics like carbapenems, colistin, and tigecycline [5,6,7,8].

Although intrinsically susceptible to most antibiotics, *E. coli* can acquire resistance genes through horizontal gene transfer, making it a significant cause of nosocomial infections and a growing public health concern [9,10,11]. As a commensal inhabitant of the gastrointestinal tract, it can serve as a reservoir for resistance genes while also being a leading pathogen responsible for urinary tract infections and sepsis [12,13,14].

β-lactam antibiotics, which include penicillin, cephalosporins, monobactams, and carbapenems, are among the most common agents used to treat bacterial infections [15,16]. β-lactam resistance is primarily caused by β-lactmases, enzymes that hydrolyze β-lactam rings and therefore inactivate the drug [17]. Based on amino acid sequence homology, β-lactamases are classified into four Ambler classes (A, B, C, and D). Classes A, C, and D are serine β-lactamases, whereas class B enzymes are metallo-β-lactamases that require zinc for activity [18]. New Delhi metallo-β-lactamase (NDM), a class B enzyme, confers resistance to nearly all β-lactams except monobactams, posing a significant clinical challenge [19,20].

The *bla*_NDM_ gene, typically located on a plasmid, enables the spread of carbapenem resistance through horizontal gene transfer [21]. A diverse set of plasmid types can serve as vectors for *bla*_NDM_, further promoting its dissemination [22,23,24,25,26]. Among *bla*_NDM_ variants, *bla*_NDM-5_ is a newly emerged carbapenemase with greater hydrolytic activity against carbapenems and cephalosporins (cefotaxime and ceftazidime), attributed to two mutations (Val88Leu and Met154Leu) compared to *bla*_NDM-1_ [27].

Recent surveillance reports have shown an increase in *E. coli* isolates carrying *bla*_NDM-5_ variants in the European Union/European Economic Area (EU/EEA) [28]. While European surveillance has noted this increase, detailed molecular characterization of NDM-producing *E. coli* at a national level, particularly in countries like Croatia, remains limited. Such data are crucial for understanding local transmission dynamics and informing infection control policies.

The aim of the study is to characterize the sequence types (ST), plasmid profiles, and *bla*_NDM_ gene variants in a collection of NDM-producing *E. coli* isolates collected over a one-year period, providing insights into the molecular epidemiology and potential dissemination pathways of carbapenem resistance in Croatia.

## 2. Materials and Methods

The Croatian Committee for Antibiotic Resistance Surveillance at the Croatian Academy of Medical Sciences has maintained a national surveillance program since 1997, covering >90% of the population. Since the beginning of the program, carbapenem-resistant Enterobacterales have been designated as alert organisms and are mandatorily reported to the Reference Centre for Antimicrobial Resistance Surveillance.

In this prospective study, NDM-positive E. coli isolates from clinical samples were collected through the national surveillance network of Croatian microbiological laboratories between 1 March 2023 and 31 March 2024, and submitted to the Reference Centre for Antimicrobial Resistance Surveillance. Metadata collected alongside the isolates included the submitting institution, patient demographics, specimen type, sampling date, and information on prior travel or residence outside Croatia.

Upon arrival, isolates were plated on blood agar, incubated overnight at 36 ± 1 °C, and stored at −80 °C in 15% glycerol stock. Prior to genomic analysis, the stored isolates were streak-plated on blood agar and incubated overnight at 36 ± 1 °C. The colonies were identified using matrix-assisted laser desorption ionization-time of flight mass spectrometry (MALDI-TOF; Bruker Diagnostics, Bremen, Germany). In-house PCR retesting was performed to confirm the presence of the carbapenemase genes, including *bla*_NDM_, *bla*_OXA-48-like_, *bla*_VIM_, *bla*_IMP_, and *bla*_KPC_.

Total DNA was extracted from the selected colonies using the ZymoBIOMICS^TM^ DNA Miniprep Kit (Zymo Research, Irvine, CA, USA) following the manufacturer’s instructions. Genomic libraries were prepared with the Nextera XT DNA Library Preparation Kit (Illumina Inc., San Diego, CA, USA). The Illumina MiSeq platform (Illumina Inc., USA) was used for the 2 × 250 bp paired-end sequencing.

For bioinformatic analysis, default parameters were used. Firstly, raw data quality was assessed with FastQC (v0.12.1) [29]. De novo genome assembly of the short reads was performed using SPAdes Genome Assembler (v3.15.2) [30]. QUAST (v5.0.2) was used to generate genome assembly statistics [31]. KmerFinder (v3.0.2) was used for silico identification of bacterial species [32,33]. Multilocus sequence typing (MLST v2.0) was used to determine STs [34]. PlasmidFinder (v2.0.1) was used for identification of plasmid incompatibility groups [35]. AMRFinderPlus (v4.0.3) was used to find acquired AMR genes and point mutations [36]. Phylogenetic reconstruction was performed using Parsnp (v2.1.0), which constructs an assembly-based alignment of core-genome single-nucleotide polymorphisms using the *E. coli* strain K-12 as a reference genome. [37]. Statistical analysis and visualization of the data were performed using R studio (V4.1.2) [38].

## 3. Results

**Figure 1:**
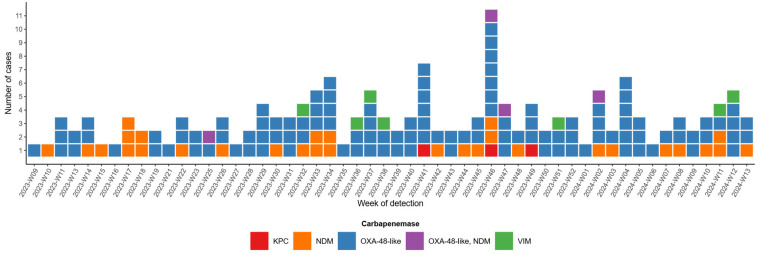
Carbapenemase-producing *E. coli* isolates in Croatia, March 2023–March 2024 (*n* = 160). Different carbapenemase types are represented with different colors, as shown in legend.

According to the national surveillance data, a total of 160 carbapenem-producing *E. coli* isolates were reported from March 2023 to the end of March 2024 in Croatia (Figure 1).

The most common carbapenemase gene was *bla*_OXA-48-like_, identified in 116 (72.50%) *E. coli* isolates, followed by *bla*_NDM_, which was detected in 30 (18.75%) isolates. The presence of dual carbapenemases (*bla*_OXA-48-like_ and *bla*_NDM_) was detected in four (2.50%) isolates. Genes *bla*_VIM_ and *bla*_KPC_ were detected in seven (4.38%) isolates and three (1.87%) isolates respectively.

Whole-genome sequencing was performed on 22 of the 34 reported isolates; the remaining isolates were unavailable as they did not survive transport or failed to grow upon subculture. Therefore, 22 NDM-producing *E. coli* isolates were carried forward for further analysis. The most prevalent *bla*_NDM_ variant was *bla*_NDM-1_, identified in 17 (77.27%) isolates, while *bla*_NDM-5_ was detected in 5 (22.73%) isolates. When comparing to other STs in this dataset, *bla*_NDM-1_ was not associated with any specific ST in this group.

**Figure 2:**
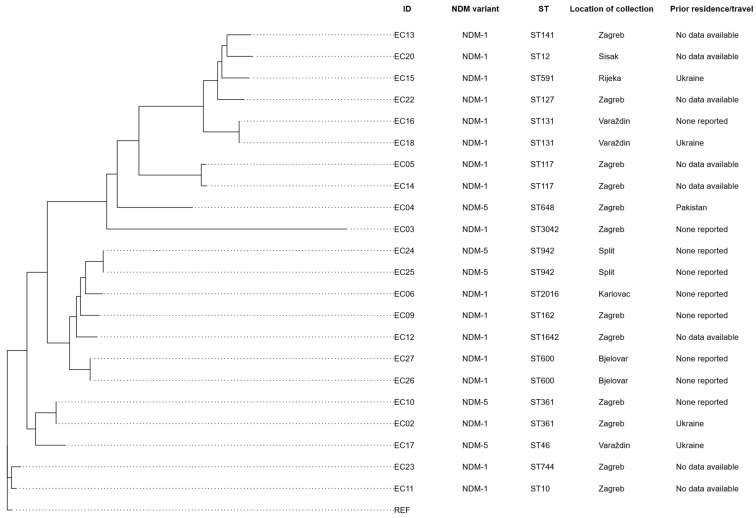
Phylogenetic analysis of NDM-producing *E. coli* isolates. The figure also includes ID, ST, NDM variant, location of collection, and prior travel or residence information within the past 6 months for each isolate.

Phylogenetic analysis was performed to investigate the relatedness among the analyzed isolates (Figure 2). The analysis revealed no significant clustering of isolates. MLST analysis revealed a total of 17 distinct STs in this dataset, highlighting considerable genetic diversity. The high level of diversity was confirmed with the Simpson diversity index, which was equal to 0.978.

Information on residence and/or prior travel within the past 6 months was available for 14 *E. coli* isolates. Among them, three (21.43%) isolates were linked to war-wounded patients from Ukraine and were associated with *bla*_NDM-1_-producing *E. coli*. Additionally, two (14.28%) isolates were associated with *bla*_NDM-5_—one from a patient who had travel history to Pakistan and one from a war-wounded patient from Ukraine. For the remaining 9 (64.29%) isolates, there were no specific data for prior travel and/or residence links. Moreover, no significant correlation was found between specific STs and prior travel and/or residence information.

**Figure 3:**
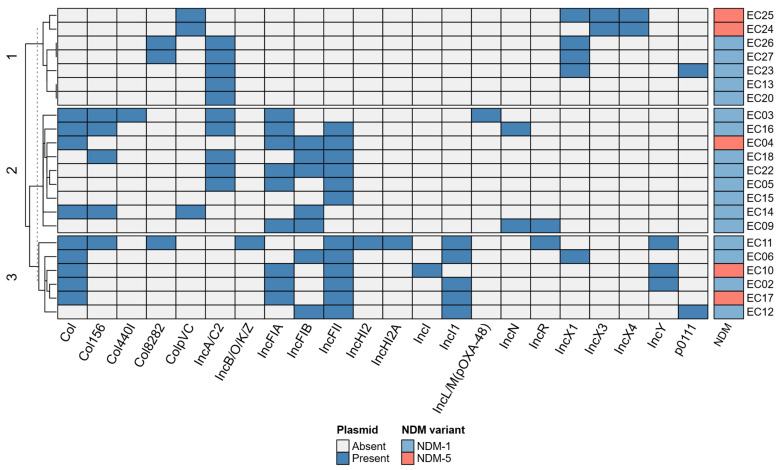
Heatmap showing plasmid replicon type diversity within analyzed isolates and its association with *bla*_NDM_ gene variants. Based on diverse profiles, samples were grouped into three clusters.

Screening of plasmid replicons among 22 *E. coli* isolates identified a diverse plasmid population with 22 replicon types. Each isolate harbored three or more plasmid replicons (Figure 3). The most frequently detected replicon type was IncFII (12/22, 54.54%), followed by IncA/C2 (10/22, 45.45%), IncFIA (9/22, 40.91%), and Col (9/22, 40.91%). Based on total replicon types, isolates carrying the *bla*_NDM-5_ were grouped into three clusters, displaying plasmid diversity within these strains.

**Figure 4:**
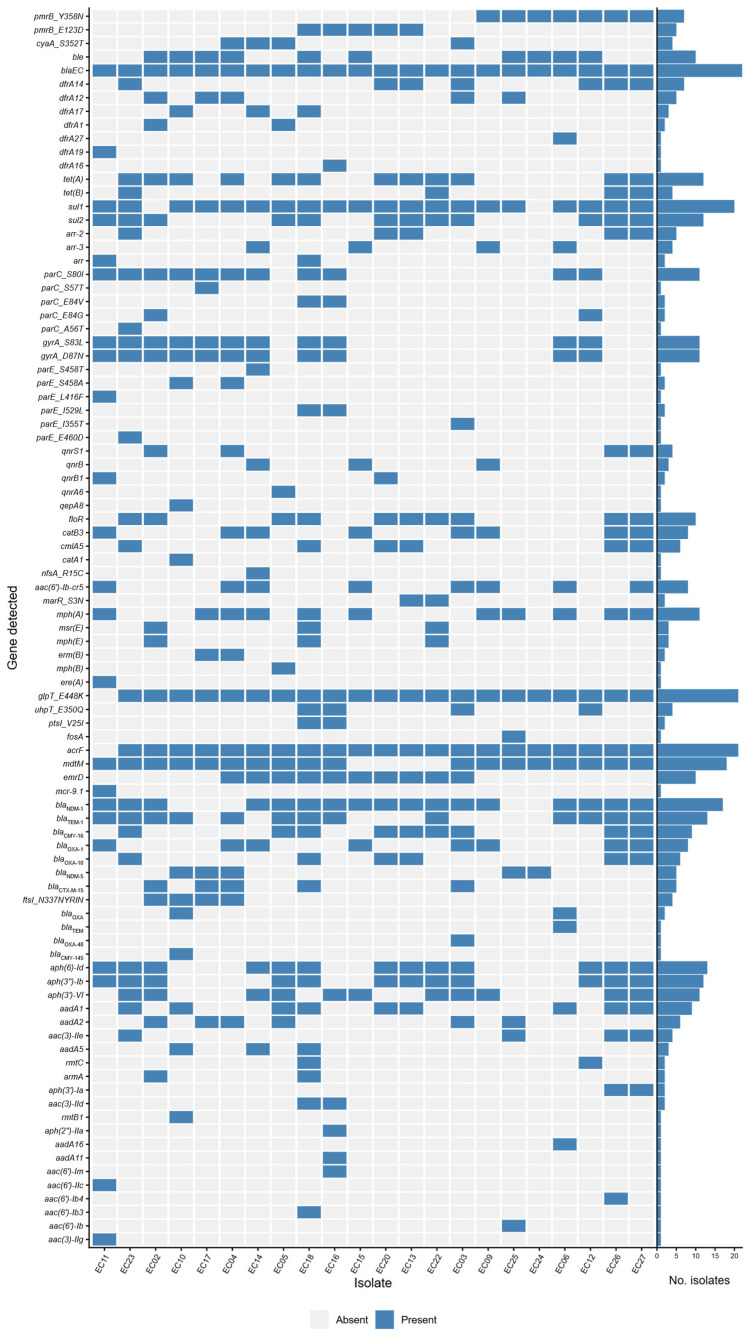
Distribution of antimicrobial resistance determinants across NDM-producing *E. coli* isolates. The heatmap illustrates the presence and absence of resistance determinants among the analyzed isolates. Additionally, a bar chart shows the cumulative number of isolates per AMR gene detected in the dataset.

The prevalence of resistance genes among the analyzed isolates is summarized in Figure 4. The isolates were screened for known resistance determinants, including both horizontally acquired genes and chromosomal point mutations.

The heatmap shows substantial variability in resistome profiles across the isolates, with no clear clustering patterns observed. While certain genes, including *bla*_NDM_, *bla*_TEM_, sulfonamide resistance genes, and efflux-associated genes (e.g., *acrF*, *mdtM*), were widely distributed, many other resistance determinants were detected sporadically. The frequent co-occurrence of multiple resistance genes across different antimicrobial classes highlights the multidrug resistance pattern across the dataset.

Notably, the combination of *bla*_NDM_ with *bla*_CTX-M-15_ and/or *bla*_CMY-16_ was detected in 12 isolates. In addition, 12 resistance determinants were present in at least 50% of the isolates, including genes associated with tetracycline resistance (*tetA*), sulfonamides (*sul1*, *sul2*), fluoroquinolones (mutations in *parC* and *gyrA*), macrolides (*mphA*), fosfomycin (mutation in *glpT*), efflux pumps (*acrF*, *mdtM*), β-lactams (*bla*_NDM-1_, *bla*_TEM-1_), and aminoglycosides (*aph(6)-Id*, *aph(3″)-Ib*, *aph(3′)-VI*).

## 4. Discussion

Carbapenem-resistant Enterobacterales are classified as critical priority pathogens by the World Health Organization (WHO) due to their association with high morbidity and mortality, posing a significant challenge to healthcare systems [39]. This study analyzed the distribution of NDM-producing *E. coli* isolates in Croatia over one year, examining their STs, plasmid replicon types, and AMR genes. Additionally, we present national surveillance data from 1 March 2023 to 31 March 2024, providing insights into trends of carbapenem-resistant *E. coli* (CREC) in Croatia.

Recent global surveillance highlights clear geographical differences in CREC epidemiology. While *bla*_NDM_ predominates worldwide, particularly in Asia and North America, European data show a different pattern, with *bla*_OXA-48-like_ as the most prevalent carbapenemase, followed by *bla_NDM_* [40]. In contrast, *bla*_KPC_ is more strongly associated with South America but is also present in parts of Europe and North America [40]. Our national surveillance data align with the detected European trend, identifying *bla*_OXA-48-like_ as the most frequent carbapenemase gene among CREC isolates in Croatia, followed by *bla*_NDM_, consistent with previous European reports [41,42,43]. Although *bla*_KPC_-producing *E. coli* are currently detected only sporadically in our national surveillance dataset, their greater dissemination potential requires continued surveillance [44].

A recent European Centre for Disease Prevention and Control (ECDC) surveillance report from 2023 highlighted an increasing prevalence of *bla*_NDM-5_ *E. coli* isolates in the EU/EEA since 2012 [28]. However, most of these isolates were linked to regions outside the EU/EEA, particularly Asia and Africa. In contrast, our dataset did not reveal a similar trend. Although we had limited information on prior travel and residence, available data linked *bla*_NDM-1_ isolates to war-wounded patients transferred from Ukraine and patients with no documented travel history, whereas *bla*_NDM-5_ isolates were predominantly identified in patients with no documented travel history, except for two cases associated with travel to Pakistan and a wounded soldier from Ukraine.

Even though the genetic diversity of NDM-producing *E. coli* isolates is high, previous studies have identified five dominant STs associated with carbapenem resistance: ST131, ST167, ST405, ST410, and ST1284 [45]. Our phylogenetic analysis confirmed a high genetic diversity among isolates, suggesting a polyclonal spread. This was further supported by ST profiling, where most isolates did not cluster closely. Five clusters of two isolates each were identified, including a cluster of ST131 isolates, as well as clusters belonging to ST942, ST600, ST117, and ST361. The ST131 cluster comprised two isolates carrying *bla*_NDM-1_. ST131 is a globally disseminated lineage commonly associated with the spread of *bla*_CTX-M-15_, and it may similarly contribute to the dissemination of carbapenemase genes [46].

According to ECDC surveillance, five dominant STs were associated with *bla*_NDM-5_ in *E. coli* isolates from Europe: ST167, ST405, ST410, ST361, and ST648 [28]. Our analysis found that Croatian isolates carrying *bla*_NDM-5_ were associated with ST361 and ST648, further supporting their role in carbapenemase gene dissemination. Additionally, ST942 was the only clonal lineage with *bla*_NDM-5_ that formed a cluster in our dataset, suggesting a potential clonal outbreak and new ST to continue to monitor.

Plasmids play a crucial role in the dissemination of *bla*_NDM_ genes, facilitating horizontal gene transfer and contributing to the rapid spread of carbapenem resistance among Gram-negative bacteria [21,47]. In this study, 22 different plasmid replicon types were identified among the analyzed isolates, with IncFII and IncA/C being the most prevalent. IncFII, a common conjugative plasmid in *E. coli*, plays a critical role in the spread of resistance genes, including *bla*_NDM-1_, among Gram-negative bacteria [48]. Similarly, the IncA/C2 plasmid family has been recognized as clinically significant due to its ability to carry and transfer multiple resistance determinants [49]. Additionally, the IncX family was associated with *bla*_NDM-5_ [50]. In our study, isolates carrying IncX replicon types formed a distinct cluster based on plasmid profile analysis, particularly associated with ST942, distinguishing them from the remaining isolates and further supporting this association.

The emergence of CREC is particularly concerning, as *E. coli* had the highest estimated incidence of invasive isolates in the EU/EEA among all reporting laboratories in 2021. According to published data from 2023, eight out of 44 EU/EEA countries (18%) reported CREC rates of 1% or higher [51]. While Croatia remains in the low-resistance category, this should be interpreted with caution, as the number of CREC isolates is increasing. Our dataset identified 76 different resistance genes, highlighting the complexity of resistance mechanisms. The combination of multiple mechanisms often results in multidrug-resistant or extensively drug-resistant strains, significantly limiting therapeutic options. Another critical concern is the high proportion of isolates co-harboring ESBL-encoding genes, rendering them resistant to monobactam. In our dataset, approximately half of NDM-positive isolates (54.55%) carried either one or both *bla*_CTX-M-15_ and *bla*_CMY-16_ genes, further limiting the effectiveness of β-lactam antibiotics. This combination has major clinical implications for treatment options. Additionally, high resistance rates to aminoglycosides, fosfomycin, and quinolones further complicate treatment, highlighting the need for surveillance.

## 5. Conclusions

This study provides insight into the molecular epidemiology of NDM-producing *E. coli* isolates from Croatia over a one-year period. Genomic characterization revealed high diversity among sequence types, with no evidence of clonal clustering. Consistent with this, a diverse range of plasmid replicon types was identified, highlighting the complexity of plasmid transmission dynamics. Importantly, the *bla*_NDM-5_ variant was detected in multiple isolates, indicating its sporadic presence in Croatia. Of particular concern is the co-occurrence of *bla*_NDM_ and ESBL-encoding genes, which significantly limits therapeutic options for β-lactam antibiotics.

These findings highlight the importance of continuous genomic surveillance at the national level to better understand local transmission dynamics and inform infection control policies. Strengthening infection prevention and control measures, alongside antimicrobial stewardship programs, is essential to limit further spread.

Our study has several limitations. It was based on a relatively small number of isolates collected over a short time, and the use of short-read sequencing restricted full plasmid characterization. Nevertheless, this study contributes valuable insights into the epidemiology of NDM-producing *E. coli* in Croatia and underscores the need for coordinated, multidisciplinary efforts to combat antimicrobial resistance.

## Data Availability

Sequence data from 22 sequenced isolates of NDM-producing *E. coli* have been deposited in the European Nucleotide Archive (ENA) database under study accession number PRJEB87695.

## References

[1] European Centre for Disease Prevention and Control, World Health Organization (2022). Antimicrobial Resistance Surveillance in Europe 2022: 2020 Data.

[2] Murray C.J.L., Ikuta K.S., Sharara F., Swetschinski L., Aguilar G.R., Gray A., Han C., Bisignano C., Rao P., Wool E. (2022). Global burden of bacterial antimicrobial resistance in 2019: A systematic analysis. Lancet.

[3] Logan L.K., Weinstein R.A. (2017). The Epidemiology of Carbapenem-Resistant Enterobacteriaceae: The Impact and Evolution of a Global Menace. J. Infect. Dis..

[4] Growing Resistance to Last-Line Antibiotics: 2013. https://antibiotic.ecdc.europa.eu/en/publications-data/growing-resistance-last-line-antibiotics-2013.

[5] Sheu C.-C., Chang Y.-T., Lin S.-Y., Chen Y.-H., Hsueh P.-R. (2019). Infections Caused by Carbapenem-Resistant Enterobacteriaceae: An Update on Therapeutic Options. Front. Microbiol..

[6] Li H., Lin H., Fu Q., Fu Y., Tan J., Wang Y., Zhou R., Sun Z., Li J. (2025). Co-transfer of mcr-1 and mcr-3 variant in *Escherichia coli* ST1632 isolate in China: Silence is not always golden. Pak. Vet. J..

[7] Haeili M., Aghajanzadeh M., Moghaddasi K., Omrani M., Ghodousi A., Cirillo D.M. (2025). Emergence of transferable tigecycline and eravacycline resistance gene tet(X4) in *Escherichia coli* isolates from Iran. Sci. Rep..

[8] Madni W., Zahoor M.A., Nawaz Z., Khurshid M. (2025). Prevalence and sequence analysis of *Escherichia coli* harboring colistin, gentamicin, streptomycin, tetracycline and quinolone resistance genes from commercial broilers. Pak. Vet. J..

[9] Mangroliya D., Adhyaru H., Kabariya J., Ramani V. (2025). Genomic insights into plasmid mediated AMR genes, virulence factors and mobile genetic elements in raw milk *Escherichia coli* from Gujarat, India. Sci. Rep..

[10] Poirel L., Madec J.-Y., Lupo A., Schink A.-K., Kieffer N., Nordmann P., Schwarz S. (2018). Antimicrobial Resistance in *Escherichia coli*. Microbiol. Spectr..

[11] Tobin E.H., Zahra F. (2026). Nosocomial Infections. StatPearls [Internet].

[12] Aziz M., Park D.E., Quinlivan V., Dimopoulos E.A., Wang Y., Sung E.H., Roberts A.L.S., Nyaboe A., Davis M.F., Casey J.A. (2025). Zoonotic *Escherichia coli* and urinary tract infections in Southern California. mBio.

[13] Ducarmon Q.R., Zwittink R.D., Willems R.P.J., Verhoeven A., Nooij S., van der Klis F.R.M., Franz E., Kool J., Giera M., Vandenbroucke-Grauls C.M.J.E. (2022). Gut colonisation by extended-spectrum β-lactamase-producing *Escherichia coli* and its association with the gut microbiome and metabolome in Dutch adults: A matched case-control study. Lancet Microbe.

[14] Wende M., Osbelt L., Eisenhard L., Lesker T.R., Damaris B.F., Mutukumarasamy U., Bielecka A., Almási É.D.H., Winter K.A., Schauer J. (2025). Suppression of gut colonization by multidrug-resistant *Escherichia coli* clinical isolates through cooperative niche exclusion. Nat. Commun..

[15] Anderson S.J., Feye K.M., Schmidt-McCormack G.R., Malovic E., Mlynarczyk G.S., Izbicki P., Arnold L.F., Jefferson M.A., de la Rosa B.M., Wehrman R.F. (2016). Off-Target drug effects resulting in altered gene expression events with epigenetic and “Quasi-Epigenetic origins”. Pharmacol. Res..

[16] Balsalobre L., Blanco A., Alarcón T., Capelo-Martínez J., Igrejas G. (2019). Beta-Lactams. Antibiotic Drug Resistance.

[17] De Angelis G., Del Giacomo P., Posteraro B., Sanguinetti M., Tumbarello M. (2020). Molecular Mechanisms, Epidemiology, and Clinical Importance of β-Lactam Resistance in Enterobacteriaceae. Int. J. Mol. Sci..

[18] Ambler R. (1980). The structure of β-lactamases. Philos. Trans. R. Soc. Lond. B Biol. Sci..

[19] Boyd S.E., Livermore D.M., Hooper D.C., Hope W.W. (2020). Metallo-β-Lactamases: Structure, Function, Epidemiology, Treatment Options, and the Development Pipeline. Antimicrob. Agents Chemother..

[20] Naas T., Oueslati S., Bonnin R.A., Dabos M.L., Zavala A., Dortet L., Retailleau P., Iorga B.I. (2017). Beta-lactamase database (BLDB)–structure and function. J. Enzym. Inhib. Med. Chem..

[21] Sakamoto N., Akeda Y., Sugawara Y., Matsumoto Y., Motooka D., Iida T., Hamada S. (2022). Role of Chromosome- and/or Plasmid-Located *bla*_NDM_ on the Carbapenem Resistance and the Gene Stability in *Escherichia coli*. Microbiol. Spectr..

[22] Sheng J., Lan H., Wang X., Yao J., Hu Y., Guo J., Zhou L., Tang X., Xu H., Yu Y. (2025). Global geographic and genomic epidemiology analysis of carbapenem-resistant *Escherichia coli* carrying *bla*_NDM-9_. mSphere.

[23] Huang Y.-S., Tsai W.-C., Li J.-J., Chen P.-Y., Wang J.-T., Chen Y.-T., Chen F.-J., Lauderdale T.-L., Chang S.-C. (2021). Increasing New Delhi metallo-β-lactamase-positive *Escherichia coli* among carbapenem non-susceptible Enterobacteriaceae in Taiwan during 2016 to 2018. Sci. Rep..

[24] Zhai Y., Lee S., Teng L., Ma Z., Hilliard N.B., May R.J., Brown S.A., Yu F., Desear K.E., Cherabuddi K. (2021). Dissemination mechanisms of NDM genes in hospitalized patients. JAC-Antimicrob. Resist..

[25] Nisa T.T., Sugawara Y., Hamaguchi S., Takeuchi D., Abe R., Kuroda E., Morita M., Zuo H., Ueda A., Nishi I. (2024). Genomic characterization of carbapenemase-producing Enterobacterales from Dhaka food markets unveils the spread of high-risk antimicrobial-resistant clones and plasmids co-carrying blaNDM and mcr-1.1. JAC Antimicrob. Resist..

[26] Weber A., Denkel L., Geffers C., Kola A., Maechler F. (2025). NDM-1 plasmid clustering reflects clonal transmission of *Klebsiella pneumoniae* ST147 in four hospitals in Berlin, Germany. Antimicrob. Resist. Infect. Control.

[27] Li L., Gao Y., Wang L., Lu F., Ji Q., Zhang Y., Yang S., Cheng P., Sun F., Qu S. (2024). The effects of NDM-5 on *Escherichia coli* and the screening of interacting proteins. Front. Microbiol..

[28] European Centre for Disease Prevention and Control (2023). Increase in Escherichia coli Isolates Carrying blaNDM-5 in the European Union/European Economic Area, 2012–2022.

[29] Andrews S. FastQC: A Quality Control Tool for High Throughput Sequence Data. https://www.bioinformatics.babraham.ac.uk/projects/fastqc/.

[30] Bankevich A., Nurk S., Antipov D., Gurevich A.A., Dvorkin M., Kulikov A.S., Lesin V.M., Nikolenko S.I., Pham S., Prjibelski A.D. (2012). SPAdes: A New Genome Assembly Algorithm and Its Applications to Single-Cell Sequencing. J. Comput. Biol..

[31] Gurevich A., Saveliev V., Vyahhi N., Tesler G. (2013). QUAST: Quality assessment tool for genome assemblies. Bioinformatics.

[32] Hasman H., Saputra D., Sicheritz-Ponten T., Lund O., Svendsen C.A., Frimodt-Møller N., Aarestrup F.M. (2014). Rapid Whole-Genome Sequencing for Detection and Characterization of Microorganisms Directly from Clinical Samples. J. Clin. Microbiol..

[33] Larsen M.V., Cosentino S., Lukjancenko O., Saputra D., Rasmussen S., Hasman H., Sicheritz-Pontén T., Aarestrup F.M., Ussery D.W., Lund O. (2014). Benchmarking of Methods for Genomic Taxonomy. J. Clin. Microbiol..

[34] Larsen M.V., Cosentino S., Rasmussen S., Friis C., Hasman H., Marvig R.L., Jelsbak L., Sicheritz-Pontéen T., Ussery D.W., Aarestrup F.M. (2012). Multilocus Sequence Typing of Total-Genome-Sequenced Bacteria. J. Clin. Microbiol..

[35] Carattoli A., Zankari E., Garcìa-Fernandez A., Larsen M., Lund O., Voldby Villa L., Møller Aarestrup F., Hasman H. (2014). In Silico Detection and Typing of Plasmids using PlasmidFinder and Plasmid Multilocus Sequence Typing. Antimicrob. Agents Chemother..

[36] Feldgarden M., Brover V., Gonzalez-Escalona N., Frye J.G., Haendiges J., Haft D.H., Hoffmann M., Pettengill J.B., Prasad A.B., Tillman G.E. (2021). AMRFinderPlus and the Reference Gene Catalog facilitate examination of the genomic links among antimicrobial resistance, stress response, and virulence. Sci. Rep..

[37] Treangen T.J., Ondov B.D., Koren S., Phillippy A.M. (2014). The Harvest suite for rapid core-genome alignment and visualization of thousands of intraspecific microbial genomes. Genome Biol..

[38] RStudio Team (2020). RStudio: Integrated Development for R.

[39] WHO (2024). Bacterial Priority Pathogens List 2024: Bacterial Pathogens of Public Health Importance, to Guide Research, Development, and Strategies to Prevent and Control Antimicrobial Resistance.

[40] Huang J., Lv C., Li M., Rahman T., Chang Y.-F., Guo X., Song Z., Zhao Y., Li Q., Ni P. (2024). Carbapenem-resistant *Escherichia coli* exhibit diverse spatiotemporal epidemiological characteristics across the globe. Commun. Biol..

[41] Findlay J., Perreten V., Poirel L., Nordmann (2022). Molecular analysis of OXA-48-producing *Escherichia coli* in Switzerland from 2019 to 2020. Eur. J. Clin. Microbiol. Infect. Dis..

[42] Peirano G., Pitout J.D.D. (2025). Rapidly spreading Enterobacterales with OXA-48-like carbapenemases. J. Clin. Microbiol..

[43] Kohlenberg A., Svartström O., Apfalter P., Hartl R., Bogaerts P., Huang T.-D., Chudejova K., Malisova L., Eisfeld J., Sandfort M. (2024). Emergence of *Escherichia coli* ST131 carrying carbapenemase genes, European Union/European Economic Area, August 2012 to May 2024. Eurosurveillance.

[44] Cao S., Jiang X., Suo J., Lu Y., Ju M., Zeng Q., Zheng Q., Zhang Z., Tang W. (2024). Molecular characteristics and antimicrobial susceptibility profiles of *bla*_KPC_-producing *Escherichia coli* isolated from a teaching hospital in Shanghai, China. Infect. Drug Resist..

[45] Peirano G., Chen L., Nobrega D., Finn T.J., Kreiswirth B.N., DeVinney R., Pitout J.D. (2022). Genomic Epidemiology of Global Carbapenemase-Producing *Escherichia coli*, 2015–2017. Emerg. Infect. Dis..

[46] Mathers A.J., Peirano G., Pitout J.D.D. (2015). The Role of Epidemic Resistance Plasmids and International High-Risk Clones in the Spread of Multidrug-Resistant Enterobacteriaceae. Clin. Microbiol. Rev..

[47] Shoaib M., Hameed M.F., Aqib A.I., Wang W., Wang Q., Wang S., Pu W. (2025). Emerging threat of antimicrobial resistance determinants and plasmid replicon types acquisition by *Escherichia coli* of poultry and other food-producing animal origin in China: Local findings with global implications. Poult. Sci..

[48] Jia Y., Hu H., Zhai Y., Zhao B., Sun H., Hu G., Pan Y., Yuan L. (2022). CpxR negatively regulates IncFII-replicon plasmid pEC011 conjugation by directly binding to multi-promoter regions. Res. Vet. Sci..

[49] Xu J., Guo H., Li L., He F. (2023). Molecular epidemiology and genomic insights into the transmission of carbapenem-resistant NDM-producing *Escherichia coli*. Comput. Struct. Biotechnol. J..

[50] Daaboul D., Kassem I.I., El Omari K., Dabboussi F., Oueslati S., Naas T., Osman M. (2024). The occurrence of the carbapenemase gene, blaNDM-5, on a transmissible IncX3 plasmid in multidrug-resistant *Escherichia coli* isolated from a farm dog. J. Glob. Antimicrob. Resist..

[51] European Centre for Disease Prevention and Control, World Health Organization (2023). Antimicrobial Resistance Surveillance in Europe 2023: 2021 Data.

